# Purifying Cytokinetic Cells from an Asynchronous Population

**DOI:** 10.1038/srep13230

**Published:** 2015-08-11

**Authors:** Einat Panet, Efrat Ozer, Tal Mashriki, Itay Lazar, Devora Itzkovich, Amit Tzur

**Affiliations:** 1 The Mina and Everard Goodman Faculty of Life Sciences, Bar-Ilan University, Ramat-Gan 5290002, Israel; 2 Advanced Materials and Nanotechnology Institute, Bar-Ilan University, Ramat-Gan 5290002, Israel

## Abstract

Cytokinesis is an intensively studied process by which the cell cytoplasm divides to produce two daughter cells. Like any other aspect of cell cycle research, the study of cytokinesis relies heavily on cell synchronization. However, the synchronization of cells during cytokinesis is challenging due to the rapid nature of this process and the shortage of cell cycle blocking agents specifically targeting this phase. Here, we demonstrate the use of standard flow cytometry for directly isolating cytokinetic cells from an asynchronous population of normally proliferating cells. This approach is based on a cell cycle marker whose temporal proteolysis, in combination with DNA quantification or cell size approximation, distinguishes cells undergoing cytokinesis. Furthermore, by avoiding doublet discrimination, typically used in flow cytometry analyses, we were able to further increase selectivity, specifically purifying cells at late cytokinesis. Our method circumvents checkpoint activation, cell cycle arrest, and any other means of pre-synchronization. These qualities, as demonstrated for both unattached and adherent cells, enable high selectivity for cytokinetic cells despite their overall low abundance in an asynchronous population. The sorted cells can then be readily used for cell biological, biochemical, and genomic applications to facilitate cytokinesis and cell cycle research.

Cell division ends with cytokinesis, a process by which a cell halves its cytoplasm in parallel with chromosome segregation and decondensation to produce two daughter cells^1^,^2^. Balanced cytokinesis is crucial for maintaining genomic integrity and indeed, canonical cytokinesis regulators are often associated with cancer and other human diseases^1^,^2^.

Cytokinesis is an intensively studied subject in cell biology. Nevertheless, the ability to obtain large quantities of late mitotic or cytokinetic cells remains a challenging bottleneck in the field. Cytokinesis is a relatively short process; consequently, the fraction of cytokinetic cells within a population of normally proliferating cells is small. In general, this limitation can be overcome by cell cycle blocking agents that pause cell cycle progression at a specific point via checkpoint mechanisms. However, not every step in the cell cycle can be directly blocked. Focusing on mitosis and cell division, there is a shortage of reagents that induce arrest after sister-chromatid separation. Even if there were such reagents, they would most likely interfere with the process of cytokinesis, thus distorting results and data interpretation.

In contrast, pre-metaphase synchronization is relatively simple, robust, and inexpensive. Microtubule polymerizing/depolymerizing agents (e.g., nocodazole and taxol), as well as kinesin inhibitors (e.g., monastrol and S-trityl-L-cysteine), interfere with mitotic spindle assembly^3^,^4^,^5^. Consequently, the metaphase plate cannot be formed, the mitotic checkpoint is activated, and cells are arrested with 4 N DNA and fully condensed chromosomes. This synchronization approach is effective; for example, nocodazole blocks cells at pre-metaphase with nearly 100% efficiency. However, effective synchronization at pre-metaphase requires prolonged exposure to chemicals that are, by definition, hazardous.

Synchronization of mammalian cells in cytokinesis (C-phase) is typically achieved by releasing cells from pre-metaphase arrest (see, for example, Ref. 6). However, pre-metaphase blockers damage cytoskeletal organization, potentially introducing unwanted variables to the upcoming cytokinesis. Moreover, cells respond differently to drugs due to i) non-genetic heterogeneity; ii) uneven cell cycle arrest resulting from the random cell cycle position of each cell before treatment; and iii) non-cell autonomous effects. No less heterogeneous is the recovery from drug arrests; for instance, in HEK293 human cells, a substantial proportion of mitotic cells is seen three hours after nocodazole removal despite the short length of mitosis (<1 h)^7^. Together, these phenomena inevitably limit the quality of synchronization, especially in processes such as cytokinesis that capture a small portion of the mammalian cell cycle.

Drug-free synchronization is inherently preferable. Biomechanical approaches for cell cycle synchronization, including centrifugal elutriation, “baby-machine”, and size-based sorting^7^,^8^,^9^,^10^, as well as serum starvation, have proven efficient for synchronization at the G1 phase. However, the cell-to-cell variability in cell cycle progression, also known as dispersion, will significantly reduce synchronization by the time cells reach mitosis^7^. Therefore, these approaches have limited use in the synchronization of cells during cytokinesis.

Cell cycle arrest at the G1-S transition (e.g., by double thymidine block) brings cells closer to cytokinesis and does not involve cytoskeletal toxicity. However, any type of cell cycle blocker may dissociate the cell cycle from cell growth in ways that can affect division input^10^. Furthermore, the combination of heterogeneous response and release from the drug with natural dispersion during S, G2, and early M phases would inevitably lower synchronization during cytokinesis. Therefore, when G1-S synchronization is used to enrich cytokinetic cells, the protocol often involves a second synchronization step in mitosis (see, for example, Ref. 11).

We have recently demonstrated the use of standard flow cytometry for synchronizing mammalian cells in G1 without blocking cell cycle progression^7^. Driven by our own need for minimally perturbed late mitotic and cytokinetic cells^12^,^13^, we have developed a cytometry-based approach for purifying cytokinetic cells directly from an asynchronous population of proliferating cells. The method is simple and robust, as demonstrated for both adherent and unattached cells.

## Results

### Isolating cytokinetic cells by cytometry

**Figure 1:**
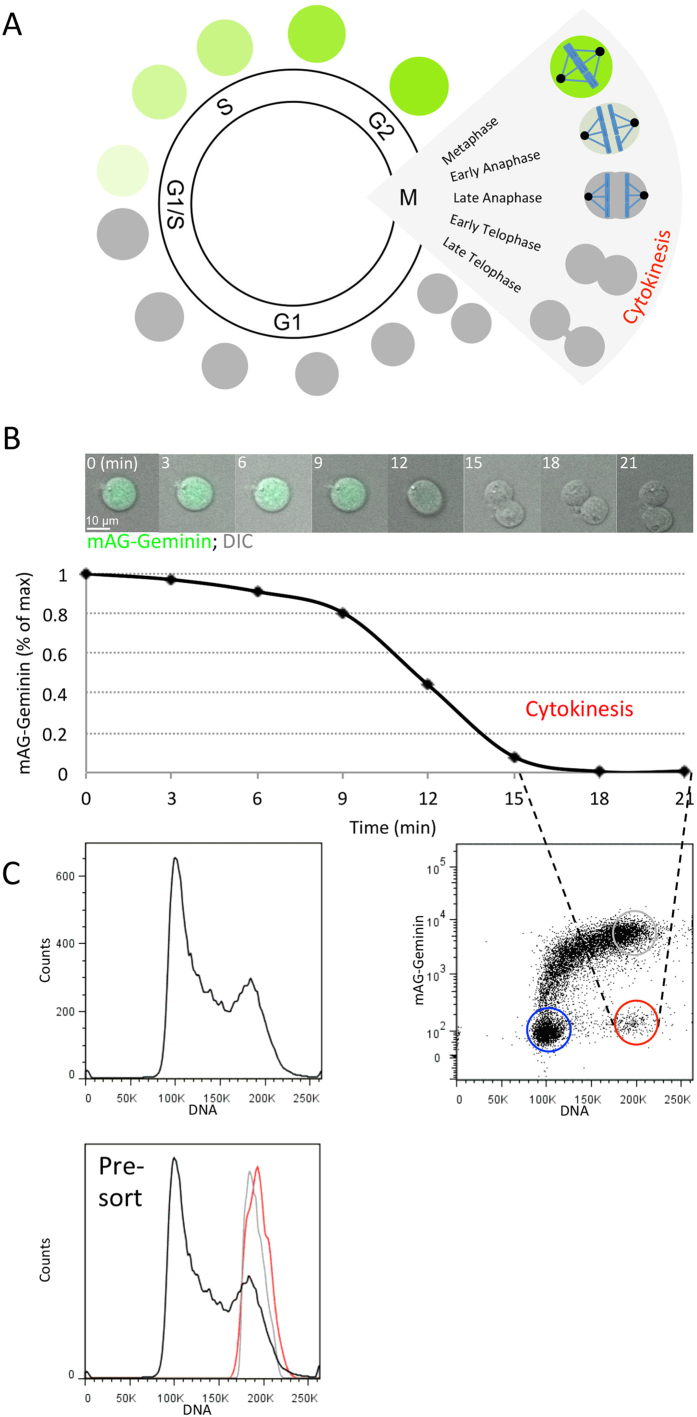
Purifying cytokinetic cells – strategy and practice. **(A)** An illustration of mAG-Geminin dynamics in proliferating cells. mAG-Geminin signal drops at the metaphase-to-anaphase transition, and starts accumulating again at the G1-S transition of the following cycle. During cytokinesis, mAG-Geminin levels are low. **(B)** mAG-Geminin dynamics in live L1210 cells. Time-lapse microscopy of a representative L1210 cell stably expressing mAG-Geminin. Images were taken at 3- min intervals using a ×20 air lens. mAG signal was normalized to maximum intensity at *t* = 0 (% of max). **(C)** (Top, left) mAG-Geminin-expressing L1210 cells were stained with Hoechst 33342, and their DNA distribution was measured by FACSAria III. (Top, right) A bivariate plot of mAG-Geminin vs. DNA. A distinct population of cells with 4 N DNA and low mAG-Geminin levels was gated (red circle). G2-early M cells and G1 cells were gated as well (gray and blue circles, respectively). (Bottom) DNA distributions of cytokinetic cells (red), G2-early M cells (gray), and the entire population (black) are shown.

Fluorescently tagged cell cycle proteins have been widely used as cell cycle markers. These fusion proteins are constitutively expressed, and their level is regulated by the ubiquitin-proteasome system, such that the markers’ temporal proteolysis indicates distinct points along the cell cycle^14^,^15^,^16^. Geminin is a target of the ubiquitin ligase anaphase-promoting complex/cyclosome (APC/C)^17^. Geminin degradation commences at the metaphase-to-anaphase transition and is maintained throughout the G1 phase. Following APC/C inactivation at the G1/S transition, Geminin accumulates, reaching a peak level at G2 and early mitosis (Fig. 1A). Geminin N-terminus carries a degradation motif (degron) that is recognized by the APC/C, but does not seem to exhibit any other activity. When fused to a fluorescent tag, this fragment mirrors APC/C activity and cell cycle progression with no apparent toxicity^15^.

Murine lymphoblastoid L1210 cells stably expressing Geminin N-terminus attached to monomeric Azami-Green (mAG), have already been described^16^. The quick disappearance of mAG-Geminin during anaphase can be seen in the image series depicted in Fig. 1B. In this representative time-lapse experiment, mAG-Geminin levels drop by ~75% within 6 min following anaphase onset. From late anaphase to daughter-cell separation, mAG-Geminin is at its lowest level, and the amount of DNA is 4 N. This two-parameter signature is unique to cells during cytokinesis because the amount of DNA is halved once division is completed. Thus, cytokinetic cells can be distinguished from both G2-early M cells (maximum mAG-Geminin levels and 4 N DNA) and G1 cells (minimum mAG-Geminin levels and 2 N DNA).

Cell sorting by optical flow cytometry is a fast and robust tool for isolating cells of interest from a heterogeneous cell population. We decided to test whether this technology can be utilized for purifying cytokinetic cells from an asynchronous population without blocking the cell cycle. We labeled the DNA of live mAG-Geminin-expressing L1210 cells (Fig. 1C, top left plot). A bivariate plot showing mAG-Geminin vs. DNA revealed a distinct, time-invariant, population of cells with nearly minimum levels of green fluorescence and 4 N DNA (Fig. 1C, top right plot). This subpopulation (red circle), which we estimated to be <1% of the entire cell population, could be easily distinguished from both G2-early M and G1 subpopulations (gray and blue circles, respectively). The plot in Fig. 1C also shows that the fraction of cells with 4 N DNA ranging between maximum and minimum mAG-Geminin levels is effectively negligible, reflecting on the rapid degradation of mAG-Geminin during the short process of anaphase (Fig. 1B). DNA distributions of putative cytokinetic cells, G2-early M cells, and the entire population are shown in Fig. 1C, bottom plot.

**Figure 2:**
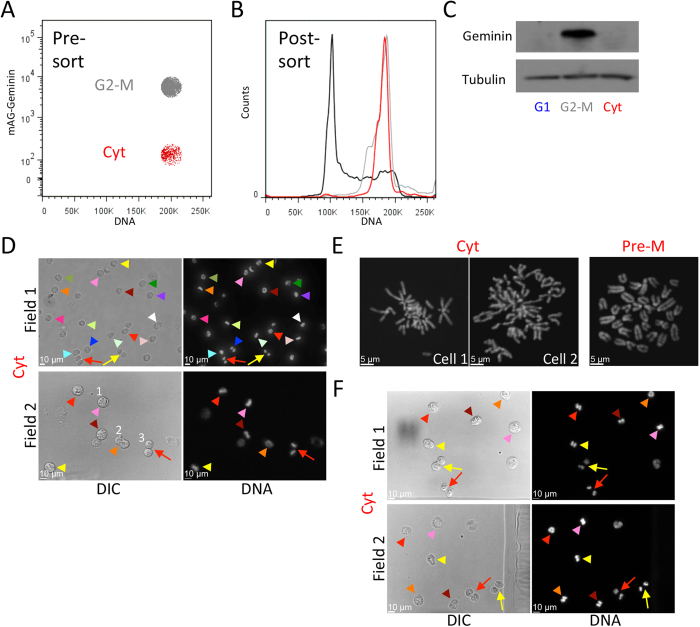
Characterization of cytokinetic L1210 cells post-sort. (**A**) A bivariate plot showing only the G2-early M (G2-M; gray) and cytokinetic (Cyt; red) L1210 cell populations gated for sorting. (**B,C**) The sorted populations were either fixed and stained with propidium iodide to determine the DNA distribution post-sort (**B**) or harvested for Western blotting with anti-Geminin and anti-Tubulin (loading control) antibodies (**C**). G1 L1210 cells were sorted based on size approximation^7^. Mean percentage of Cyt cells with 4 N DNA was calculated from 3 independent experiments; standard deviation [SD] = 4.3. (**D,F**) mAG-Geminin-expressing L1210 cells were labeled with Hoechst 33342. The Cyt subpopulation was sorted into glass-bottom dishes filled with 4% paraformaldehyde (PFA) solution (**D**). Alternatively, DNA-labeled cells were first fixed (PFA), washed in PBS, and then gated for sorting and imaging (**F**). Cells were imaged with ×20 or ×40 lenses using DIC and 445/50 nm excitations for monitoring cell shape and chromatin morphology, respectively. Images of two representative fields of view are shown. Arrowheads indicate early cytokinetic cells for which condensed chromatin can be observed. Arrows indicate late cytokinetic cells. Percentages of 8-shaped cells post-sort were calculated from 380 (**D**) and 413 (**F**) cells. **(E)** Cytokinetic cells were sorted directly into a hypotonic buffer, spread on glass slides, mounted, and imaged by a ×100 oil lens. Chromosome spreads of two representative cells are shown. A chromosome spread of a pre-metaphase cell (Pre-M) arrested by nocodazole block is shown for purposes of comparison.

The gated cytokinetic (Cyt) and G2-early M (G2-M) cell populations were sorted for further analyses (Fig. 2A). First, we examined DNA distribution post-sort. We used propidium iodide staining, which is thought to quantify DNA more accurately than live-cell DNA dyes. As can be seen in Fig. 2B, 92% of the cells that were marked as cytokinetic pre-sort contained 4 N DNA, indicating that they did not yet complete division. DNA distributions of G2-early M cells and the entire cell population (post-sort) are shown for comparison. As expected, the mAG-Geminin level in the sorted Cyt population was two orders of magnitude lower than in the G2-early M population (Fig. S1). Sorted cytokinetic and G2-early M cells were also processed for Western blotting. In addition, we collected G1 cells according to our recently published sorting protocol^7^. Western blot analysis with an antibody against endogenous Geminin showed a bright signal in G2-early M cells. This signal disappeared in both cytokinetic and G1 cells in accordance with mAG-Geminin dynamics during mitotic exit.

Next, we examined the cellular and chromatin morphology of the Cyt population. Cells were sorted directly into optical transparent dishes filled with a fixative solution in order to minimize cell cycle progression post-sort. Images of two representative optical fields of view are shown in Fig. 2D. The majority of the sorted cells appeared at various mitotic stages, as demonstrated by their condensed chromatin (marked by arrowheads) and cellular morphology (see, for example, the numbered cells in Fig. 2D, Field 2). Although, for some cells, chromosomes may appear at the metaphase plate, it is important to emphasize that anaphase had commenced in all cells, otherwise their mAG-Geminin level would have remained at the maximum level (Fig. 1A). Chromosome spread confirmed that sister chromatids were already separated in sorted cells with condensed chromatin [see representative images in Fig. 2E and a chromosome spread of a pre-metaphase cell (Pre-M) for comparison]. Taken together, the results in Figs 1 and 2 demonstrate a selective method for isolating L1210 cells during division. We were pleased to find that the method is compatible with fixed cells (Fig. 2F and S2). This feature is valuable when cells cannot be sorted fresh, and can also provide an opportunity to sort cells after immunofluorescence labeling.

**Figure 3:**
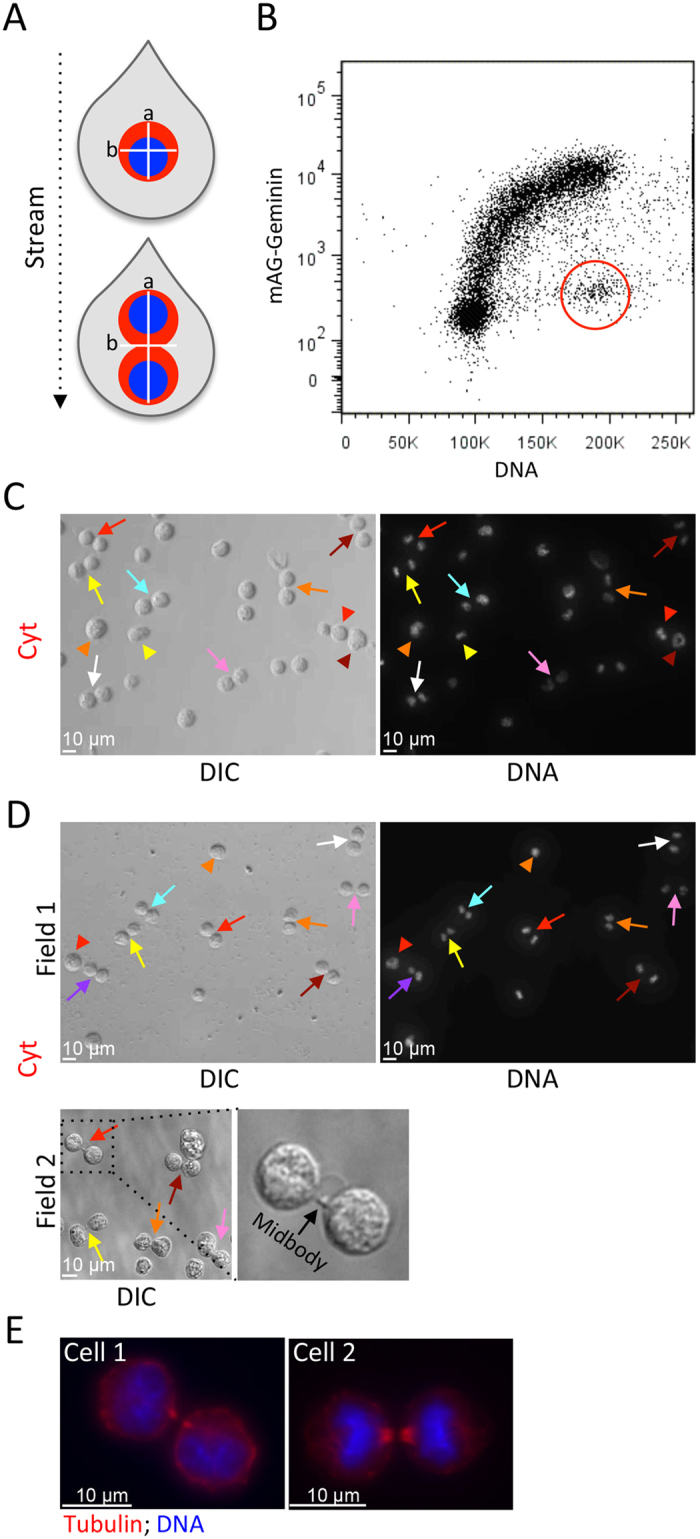
‘Doublet indiscrimination’ as a means of isolating late cytokinetic cells. (**A**) A schematic showing a stream of two droplets, each occupied by one cell. The bottom drop carries a cell at late cytokinesis. The cell appears as two adjacent cells (doublet) and should be discarded by doublet discrimination. (**B**) mAG-Geminin-expressing L1210 cells were labeled with Hoechst 33342 and analyzed by FACSAria III. A mAG-Geminin/DNA bivariate plot without doublet discrimination is depicted. Putative cytokinetic cells were gated (red). (**C,D**) The gated cells were sorted into PFA-containing dishes (**C**) or, alternatively, fixed and then sorted into PBS-containing dishes (**D**). Representative DIC and fluorescent images are shown. Arrows indicate late cytokinetic cells, some with a noticeable midbody (see magnified area in D, Field 2). Arrowheads indicate early cytokinetic cells for which condensed chromatin could be observed. Percentages of 8-shaped cells post-sort were calculated from 373 (**C**) and 390 (**D**) cells. (**E**) mAG-Geminin-expressing L1210 cells were stained with Hoechst 33342, fixed (PFA), and immunolabeled with anti-Tubulin and fluorescent secondary antibodies. Cytokinetic immunolabeled cells were processed for sorting into a glass-bottom dish, and imaged by a ×100 oil lens. Two representative cells are shown.

Less than 9% of the Cyt populations, sorted live or fixed, showed the characteristic 8-shaped morphology of cells during advanced cytokinesis (see, for example, cell #3 in Fig. 2D, Field 2). This fraction was less than we expected, considering the dynamics of mAG-Geminin relative to the cleavage furrow progression in L1210 (Fig. 1B). We hypothesized that by gating out doublets, as generally recommended in flow cytometry, we effectively excluded 8-shaped cells, defined here as late cytokinetic cells. This concern is heightened for L1210 cells, which are nearly spherical, with an average axial ratio (a/b) of 1.01^10^. Thus, the axial ratio of late cytokinetic L1210 cells is nearly 2, which is the expected axial ratio of two adjacent spherical cells considered as one particle (Fig. 3A, top vs. bottom droplet). Although doublet inclusion increases the risk of false sorting, L1210 cells naturally grow as singlets (Fig. S3), thus, doublet occurrence in these cells is expected to be low. In accordance with our expectations, doublet discrimination had a minimal impact on the overall shape of the mAG-Geminin/DNA bivariate plot (Fig. 1B vs. Fig. 3B). This sorting strategy, however, increased the fraction of 8-shaped cells sorted live or fixed to 31% and 36%, respectively [demonstrated by morphology (Fig. 3C,D)]. For some cells, the intercellular bridge and the midbody connecting the two emerging daughter cells^12^,^13^ were noticeable by differential interference contrast (DIC) microscopy (see magnified cells in Fig. 3D) and Tubulin labeling by immunofluorescence (Fig. 3E).

Together, the results shown in Figs 1, 2, 3 demonstrate a method for isolating cytokinetic cells directly from asynchronous populations of live or fixed cells. These results also show the legitimate use of ‘doublet indiscrimination’ as means for collecting late cytokinetic cells.

### Isolating cytokinetic cells without chemical labeling

DNA labeling typically relies on DNA intercalating/binding agents that must be avoided in certain cases due to their cytotoxicity. This limitation motivated us to examine alternative approaches for purifying cytokinetic cells without using DNA dyes or any other chemicals.

**Figure 4:**
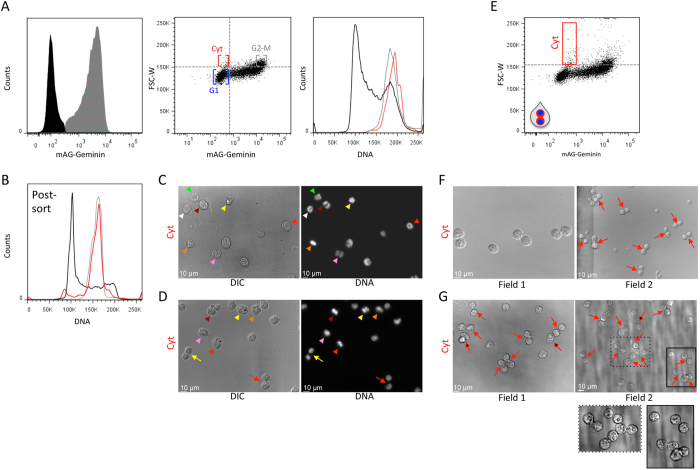
Purifying cytokinetic cells without chemical labeling. (**A**) (Left plot) A histogram showing the bimodal distribution of mAG-Geminin in L1210 cells. Cells expressing a basal mAG-Geminin level (black) are at G1 and late mitosis, and can be distinguished from the rest of the population (gray). (Mid plot) A bivariate plot showing mAG-Geminin vs. FSC-W. Large cells (high FSC-W) with low mAG-Geminin levels are framed (red); these are putative cytokinetic cells (Cyt). Large cells (high FSC-W) with high mAG-Geminin levels (gray) are putative G2 and early M cells (G2-M). Small cells (low FSC-W) with low mAG-Geminin (blue) are putative G1 cells. (Right plot) DNA distributions of gated Cyt and G2-M populations are shown in matching colors. The DNA distribution of the entire population is shown in black. (**B**) L1210 cells were gated and sorted following the mAG-Geminin/FSC-W gating strategy (A, mid plot). The purified cells were fixed and stained with propidium iodide. DNA distributions of Cyt (red), G2-M (gray), and the entire population (black) post-sort are shown. Mean percentage of Cyt cells with 4 N DNA was calculated from 3 independent experiments; SD = 7.58. (**C,D**) Cytokinetic cells were sorted into PFA-containing dishes and processed for imaging (**C**) or, alternatively, fixed and then sorted into PBS-containing dishes using the same gating strategy (**D**). Representative DIC and fluorescent images of sorted cytokinetic cells are shown. Arrowheads indicate cells at early cytokinesis identified by chromatin morphology. Arrows indicate cells at late cytokinesis identified by 8-shaped morphology. In order to simplify chromatin visualization post-sort, cells were pre-labeled with Hoechst 33342 but sorted based on FSC-W and mAG-Geminin signals only. (**E–G**) Cytokinetic cells were purified by FSC-W and mAG-Geminin (E, red rectangle) without doublet discrimination (marked by the two-cell droplet). The sorted cells were fixed (PFA) and processed for DIC imaging (**F**). A similar sorting strategy was performed on pre-fixed cells (**G**). Images of two representative fields are shown (**F,G**). Percentages of 8-shaped cells post-sort were calculated from 522 (**F**) and 470 (**G**) cells. Arrows indicate late cytokinetic cells. Asterisked arrows indicate late cytokinetic cells whose emerging daughter cells appeared at two focal planes. Framed areas in Field 2 were magnified for better visualization.

A proliferating cell doubles its size throughout its life cycle. Thus, on the average, cytokinetic cells are twice the size of newborn cells at the early G1 phase^10^,^16^,^18^. Cell size cannot be measured directly by standard cytometers, but can be approximated by light scattering; more specifically, by the pulse width of forward scatter (FSC-W) or the pulse area of side scatter (SSC-A) available in all cytometers^7^,^19^. Size approximation by light scatter is more accurate for uniform and symmetric objects and, thus, expected to be optimal for L1210 cells^19^. Cytokinetic and G1 cells can be easily distinguished from cells at the S, G2, and early M phases using the mAG-Geminin marker (see Fig. 4A, left plot, black vs. gray distributions). Within the two cell populations, cytokinetic cells are the largest, on the average, while G1 cells are the smallest. If this relatively large difference in size is apparent by light scattering, then cytokinetic cells could be potentially distinguished from G1 cells by size approximation rather than DNA content. The mAG-Geminin/FSC-W bivariate plot shows that the subset of cells with the largest size and minimal mAG-Geminin levels, although small, is noticeable pre- and post-fixation and can be distinguished from G1 cells (Fig. 4A, mid plot, Cyt, and Fig. S4). Cells with a similar size range but with high levels of mAG-Geminin are expected to be in G2 and early M (G2-M). DNA distribution of the gated Cyt and G2-M populations showed 4 N DNA pre-sort (Fig. 4A, right plot), confirming that the largest cells with low mAG-Geminin have yet to divide. Using the FSC-W/mAG-Geminin gating strategy, we sorted putative cytokinetic cells for propidium iodide staining (Fig. 4B). Seventy-eight percent of the cells had 4 N DNA post-sort. DNA distributions of G2-M cells (high FSC-W and mAG-Geminin signals) and the entire cell population are also shown. Cellular and chromatin morphology confirmed that the majority of the sorted cells were at various stages of cytokinesis (Fig. 4C). This sorting strategy was also found to be compatible with fixed cells (Fig. 4D).

In accordance with what we obtained with the initial DNA/mAG-Geminin sorting protocol (Fig. 2D,F), the fraction of late cytokinetic cells isolated by size was below 10%, which was lower than expected (Fig. 4C,D). Knowing the impact of doublet discrimination on sorting cytokinetic L1210 cells (Fig. 3), we repeated the experiment without doublet discrimination (gating strategy is shown in Fig. 4E). This change increased the fraction of late cytokinetic cells, sorted live or fixed, to 42% and 35% respectively (Fig. 4F and G vs. Fig. 4C,D). Altogether, the results shown in Fig. 4 demonstrate a method for isolating cytokinetic cells without cell cycle arrest and chemical labeling.

### Extending the method to adherent cells

With respect to cell division and cytokinesis research, adherent cells represent a more popular model than unattached cells. We, therefore, decided to test whether our method is compatible with adherent cells using HeLa epithelial human cells as proof of concept. The challenge of sorting adherent cells is greater; these cells are typically larger than lymphoblastoid cells, are more fragile, amorphous, and tend to cluster. Such cellular properties can reduce accuracy, especially in the measurement of DNA or cell size, whose dynamic range in a proliferating cell is only two-fold. Because the target population is minute, any inaccuracy may dramatically reduce purity.

**Figure 5:**
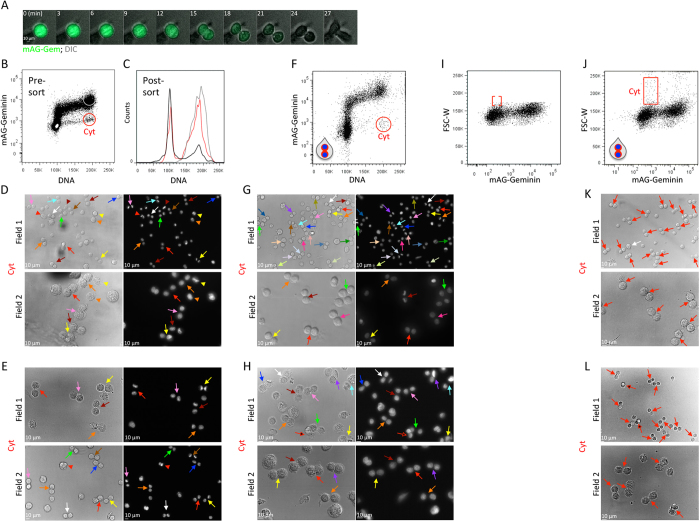
Purifying cytokinetic HeLa cells. (**A**) Time-lapse microscopy of a representative HeLa cell constitutively expressing mAG-Geminin. Images were taken at 3-min intervals using a ×20 air lens. (**B**) A bivariate plot showing mAG-Geminin vs. DNA in HeLa cells pre-labeled with Hoechst 33342. The putative Cyt and G2-early M populations are marked in red and gray, respectively. (**C**) The Cyt and G2-M populations were sorted, fixed, and stained with propidium iodide. DNA distributions are shown in matching colors. The DNA distribution of the entire population is shown in black. Mean percentage of Cyt cells with 4 N DNA was calculated from 3 independent experiments; SD = 7.87. **(D,E)** The Cyt population was sorted into PFA (**D**) or, alternatively, fixed and then sorted (**E**). DIC and fluorescent images of the sorted cells are shown. Percentages of 8-shaped cells post-sort were calculated from 330 (**D**) and 422 (**E**) cells. (**F–H**) A bivariate plot of mAG-Geminin-expressing HeLa cells labeled with Hoechst 33342 without doublet discrimination. The Cyt population was gated (red) (**F**). Live (**G**) or fixed (**H**) cytokinetic cells were sorted for imaging following the protocol described in (**F**). DIC and fluorescent images of the sorted cells are shown. Percentages of 8-shaped cells post-sort were calculated from 413 (**G**) and 470 (**H**) cells. (**I,J**) The bivariate mAG-Geminin/FSC-W plots of HeLa cells with (**I**) or without (**J**) doublet discrimination. Putative cytokinetic cells are framed (red). (**K,L**) Live (**K**) and fixed (**L**) cytokinesis HeLa cells were sorted following the protocol described in (**J**). DIC images of the sorted cells are shown. Percentages of 8-shaped cells post-sort were calculated from 358 (**K**) and 353 (**L**) cells. Two representative fields are shown for each sorting strategy. Red arrows indicate late cytokinetic cells. Asterisked red arrows indicate late cytokinetic cells whose emerging daughter cells appeared at two focal planes. The white arrow indicates a large spherical cell.

HeLa cells stably expressing mAG-Geminin markers have been successfully integrated into cell cycle research^15^,^20^. The dynamics of mAG-Geminin in a dividing HeLa cell is presented in Fig. 5A. Cytometric analysis of both live and fixed HeLa cells revealed a distinct subpopulation with 4 N DNA and low mAG-Geminin levels that, similar to L1210 cells, could be easily gated for sorting (Fig. 5B and S5). Propidium iodide staining revealed 4 N DNA for 83% of the sorted cells, meaning that the majority of cells in the Cyt population were post-anaphase, but yet to divide (Fig. 5C, red histogram). Cellular and chromatin morphology of cells, sorted live or fixed, demonstrated the method’s selectivity for cells at various stages of cytokinesis (Fig. 5D,E). In contrast to L1210 cells, this protocol also isolated late cytokinetic cells; the percentage of 8-shaped cells sorted live or fixed was 25% and 29%, respectively (marked by arrows; early cytokinetic cells are marked by arrowheads), even though doublets were discriminated (see more details in Fig. 3). These results were particularly gratifying because of our initial assumption that doublets in adherent cells are frequent and must be excluded; otherwise the small Cyt subpopulation would be heavily contaminated with doublets of G1 cells. This assumption was proven wrong; sorting live or fixed HeLa cells without doublet discrimination increased selectivity for late cytokinetic cells to over 50% (see matching-color arrows at the representative fields of view depicted in Fig. 5G,H).

Considering that cell size approximation by light scattering is less accurate in adherent cells (Fig. S6 vs. Ref. 19), we were skeptical that this metric could be used for isolating cytokinetic HeLa cells. However, the positive correlation of FSC-W (cell size) with mAG-Geminin (cell cycle progression) suggested that the limited accuracy of size approximation might still be sufficient for our purposes. In practice, the Cyt population was difficult to distinguish; thus, we avoided sorting by this gating strategy (Fig. 5I, red gate; see Fig. 4B, mid panel, for comparison). Nevertheless, we repeated the analysis without gating out doublets (Fig. 5J). To our surprise, this gating protocol was highly selectivity for late cytokinetic cells sorted live or fixed; 58% and 64%, respectively (Fig. 5K,L, marked by red arrows). The relatively small and even size of the two cells in each cell-pair suggests that these are two emerging daughter cells rather than random doublets (a singular cell is depicted for purposes of comparison; Fig. 5K, white arrow). Taken together, our methods for isolating cytokinetic cells are compatible with both unattached and adherent cells.

## Discussion

In this study, we developed a method for directly isolating cytokinetic cells from an asynchronous population of proliferating cells. We described several protocols, all of which are based on standard flow cytometry and a fluorescent marker that is quickly degraded at the metaphase-to-anaphase transition and remains low throughout G1. This marker, in combination with DNA quantification, allowed us to distinguish cells undergoing cytokinesis from the rest of the population. The selectivity of our method could be noticed by the 4 N DNA content measured for the vast majority of the sorted cells (Figs 2B and 5C), the disappearance of endogenous Geminin, as observed by bulk measurements (Fig. 2C), and the morphology of the sorted cells (Figs 2,3 and 5). Inevitably, this protocol also selects binucleated G1 cells [low mAG-Geminin level (G1) and 4 N DNA (two nuclei)]. These cells, however, emerge from spontaneous endoreduplication and, thus, are expected to be rare in normally growing cultures.

Our initial sorting strategy, performed on L1210 cells, showed a preference for early cytokinetic cells with spherical or ellipsoid morphology. This result was unexpected considering the dynamics of furrow ingression in these cells (Figs 1 and 2). We found that doublet discrimination effectively gated out 8-shaped cytokinetic cells, probably because of their appearance as doublets (Fig. 3). In previous flow cytometric analyses, we had never considered avoiding doublet discrimination. However, in both live and fixed L1210 cells, doublet discrimination was proven to be a legitimate means for distinguishing between oval cells at early cytokinesis (Fig. 2) and 8-shaped cells at late cytokinesis (Fig. 3). This sorting strategy also triples the amount of gated cells and, consequently, the sorting yield. The exclusion of 8-shaped cells by doublet discrimination was found to be less profound in HeLa (Fig. 5D,E). This difference between L1210 and HeLa cells can be explained by the sorter’s difficulty in distinguishing doublets from singlets in non-spherical objects whose light scatter is less informative with respect to cell dimensions (Fig. 3 and S6). There could also be a biological explanation for this phenomenon; if in Hela cells, mAG-Geminin signal decreases less rapidly relative to furrow ingression, then cells with minimal mAG-Geminin levels and 4 N DNA are, on the average, at a more advanced stage of cytokinesis compared to L1210 cells. Consequently, the frequency of 8-shaped cells should be higher. That being said, doublet inclusion improved selectivity for late cytokinetic cells also in HeLa (Fig. 5). This observation contradicted our long-term belief that doublets in adherent cells are frequent and must be avoided when sorting rare populations (Fig. 5F–H).

Overall, the enrichment of late cytokinetic cells resulting from doublet inclusion was profound; we estimated that more than 70% of the L1210 cells with mitotic/cytokinetic appearance post-sort were 8-shaped. This enrichment was even higher for HeLa cells. Doublet inclusion also yielded a larger fraction of small singular cells (see, for example, unmarked singular cells in Fig. 5K). While some of these cells could simply be the result of sorting ‘real’ doublets, we suspect that, for the most part, these are newly formed daughter cells that were detached from each other during or immediately after exiting the flow cell of the FACSAria III.

We evaluated the use of cell size approximation as an alternative to DNA quantification, aiming for a simpler and less hazardous protocol. Initially, we analyzed L1210 cells for which cell size approximation was expected to be optimal^19^. This sorting strategy was selective for cytokinetic cells (Fig. 4), and the impact of doublet discrimination was similar to that observed using DNA labeling (Figs 3 and 4). We did not expect sorting strategy based on size approximation to be effective in HeLa cells (Fig. S6), as was indeed shown when doublets were excluded (Fig. 5I). However, this assumption was proven incorrect when doublets were included; in fact, the selectivity for late cytokinetic HeLa cells sorted by FSC-W without doublet discrimination was better than any of the sorting strategies applied to L1210 cells (Fig. 5J–L). Thus, in cases where DNA labeling should be avoided, cytokinetic cells can be purified live or fixed by size approximation and mAG-Geminin intensity. We would, nevertheless, like to stress that size approximation by standard cytometry is not trivial, especially for adherent cells. Thus, before considering this sorting approach, cell size approximation must be carefully optimized for the desired cell type^7^,^19^.

All of our sorting strategies are expected to be selective for any type of cells for which a discrete population with low mAG-Geminin signal and either 4 N DNA or large cell size can be detected by cytometry. In this report, we used Geminin degron attached to mAG. However, the method is not limited to this specific cell cycle marker; degrons of other APC/C targets with similar temporal proteolysis (e.g., Cyclin B1 and Securin^14^), in combination with a large panel of fluorescence proteins, are likely to be equally informative. The dependence on stably transfected cells should not be an obstacle because neither Geminin degron^15^,^16^ nor the other above-mentioned APC/C degrons have apparent toxicity^14^,^21^. Moreover, mice stably expressing mAG-Geminin are viable and available commercially^15^. Thus, our approach can be readily extended to primary cells from mice and other animal models.

Currently, our protocols yield 10^2^–10^3^ cytokinetic cells per minute. Within a reasonable amount of sorting time, enough cells can be collected for cell biological and genomic applications, as well as for some proteomic and biochemical applications. Because cytokinetic cells are rare, this yield cannot be improved much unless sorting follows a preliminary enrichment of G2-M cells, preferably achieved by non-hazardous means (e.g., mitotic shake-off and release from serum deprivation). At this juncture, we would like to point out that sorters without a flow cell are more suitable for high-speed sorting, and that yield optimization was not the focus of this research.

Overall, our study describes a selective method for obtaining cytokinetic cells. The method relies on standard technology, and is independent of drugs, cell cycle arrest and chemical labeling. These qualities can facilitate cell division and cytokinesis research.

## Materials and Methods

### Cell culture

L1210 and HeLa cells were cultured in Leibovitz’s L-15 medium (Gibco; 21083-027) and Dulbecco’s Modified Eagle’s Medium (DMEM) (Biological Industries; 010551), respectively. Media were supplemented with 10% fetal bovine serum (FBS) (Biological Industries; 040071), 100 units/mL penicillin, and 100 μg/mL streptomycin (Gibco; 15140122). DMEM and Leibovitz’s L-15 were also supplemented with 1.8 mM L-Glutamine (Gibco; 25030024) and 4 g/L D-glucose solution (Sigma Aldrich; G8769), respectively. Cells were cultured at 37 ^o^C and 5% CO_2_ environment. L1210 and HeLa cells stably expressing mAG-Geminin were previously described^14^,^16^.

### Microscopy

Time-lapse experiments were conducted using a Zeiss Observer Z1 microscope equipped with a SOLA light engine® (Lumencor, Inc.) and ×20 air lens, at a normal culture environment. This microscope apparatus was also used to image sorted cells with ×20 or ×40 air lenses and a ×100 oil lens. Chromosome spreads were documented by Zeiss Axioimager Z1 microscope equipped with a ×100 oil lens. Zen (Zeiss) and ImageJ (Fiji) software were used for image processing.

### DNA staining

We used 5 μg/ml Hoechst 33342 solution (Sigma; B2661) and 20 μg/ml propidium iodide solution (Sigma Aldrich; P4170) for DNA quantification following standard labeling protocols.

### Flow cytometry

BD FACSAria III cell sorter was used throughout this study. For quantifying DNA in live cells, we used a 405 nm laser for excitation and a 450/50 nm bandpass filter for detection. For quantifying DNA by propidium iodide, we used a 561 nm laser for excitation and a 580/15 nm bandpass filter for detection. For quantifying mAG-Geminin, we used a 488 nm laser for excitation and a 530/30 nm bandpass filter for emission. Data were processed and analyzed using FlowJo v7.6 software. The 488 nm laser was used for light scattering (instrument default).

### Western blotting and immunofluorescence

L1210 cells were harvested for Western blotting following standard procedure. The following antibodies were used: anti-Geminin (Santa Cruz; SC-13015), anti-Tubulin (Neomarkers; MS581P1), and matching HRP-coupled secondary antibodies (Jackson ImmunoResearch; 115035062 and 111035144). Immunofluorescence experiments were performed as follows: mAG-Geminin expressing L1210 cells were stained with Hoechst 33342, fixed (4% PFA), and labeled with anti-α-Tubulin primary antibodies (DSHB; #12G10) and Alexa Fluor® 594-coupled goat anti-mouse secondary antibodies (Jackson ImmunoResearch Laboratories, Inc.; #115-585-003). The immunolabeled cells were processed for sorting into glass-bottomed dishes.

### Chromosome spread

Cytokinetic L1210 cells were lysed in hypotonic solution (0.8% KCl, 10 min at room temperature) and fixed (methanol/glacial acetic acid at a 3:1 ratio). Cell droplets were released from a one-meter height onto glass slides. The slides were dried and mounted with a mounting solution (Richard-Allen Scientific; 9990402). Chromosome spread of pre-metaphase cells was performed following 14 h incubation with 50 nM nocodazole (Santa Cruz Biotechnology; SC-3518).

## Additional Information

**How to cite this article**: Panet, E. *et al.* Purifying Cytokinetic Cells from an Asynchronous Population. *Sci. Rep.* **5**, 13230; doi: 10.1038/srep13230 (2015).

## Supplementary Material

Supplementary Information
