# Supplementary material for: Purifying Cytokinetic Cells from an Asynchronous Population
Source: Sci Rep. 2015 Aug 11;5:13230. doi: 10.1038/srep13230 (PMC4531312; doi:10.1038/srep13230)
Supplement: Supplementary Information [file srep13230-s1.pdf]

Supplementary Information

for

Purifying Cytokinetic Cells from an Asynchronous Population

Einat Panet[1,2]; Efrat Ozer[1,2]; Tal Mashriki[1,2]; Itay Lazar[1]; Devora Itzkovich[1];

Amit Tzur[1,2,*]

[1]The Mina and Everard Goodman Faculty of Life Sciences, Bar-Ilan University,

Ramat-Gan 5290002, Israel

[2]Advanced Materials and Nanotechnology Institute, Bar-Ilan University,

Ramat-Gan 5290002, Israel

[*]To whom correspondence should be addressed: amit.tzur@biu.ac.il

Supplementary figures and figure legends

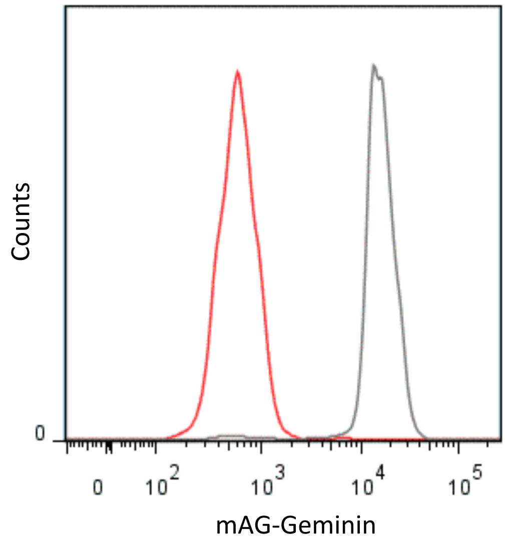

Supplementary Figure 1. ***Characterizing cytokinetic cells post-sort.*** mAG-Geminin-expressing L1210 cells were labeled with Hoechst 33342. Cyt (red) and G2-early M (gray) subpopulations were sorted (Fig. 2A) and reanalyzed by FACSAria III to quantify mAG-Geminin post-sort.

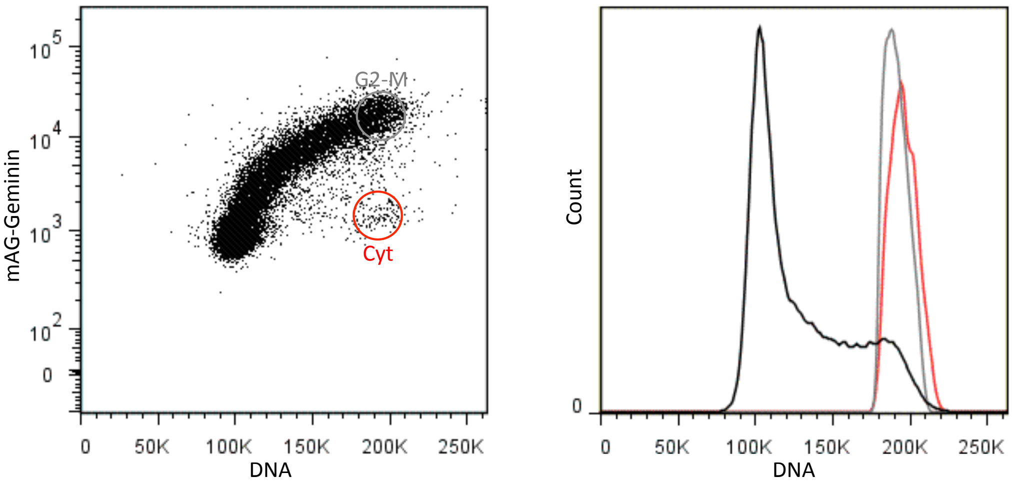

Supplementary Figure 2. ***Gating and sorting fixed cytokinetic L1210 cells.*** **(A)** L1210 cells expressing mAG-Geminin were stained with Hoechst 33342 and fixed in 4% PFA. A bivariate plot showing mAG-Geminin vs. DNA is shown. Cells with 4N DNA and low mAG-Geminin levels, i.e., the Cyt population, were gated (red). G2-early M cells (G2-M) are also indicated (gray circle). **(B)** DNA distributions of Cyt (red), G2-M (gray), and the entire populations (black) are shown.

L1210 cells

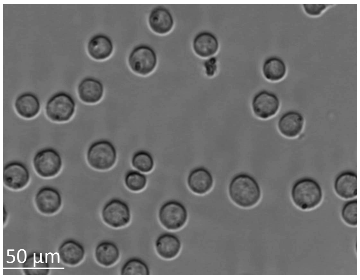

Supplementary Figure 3. ***L1210 cells grow naturally as singlets.*** A representative DIC image of L1210 cells (x20 air lens).

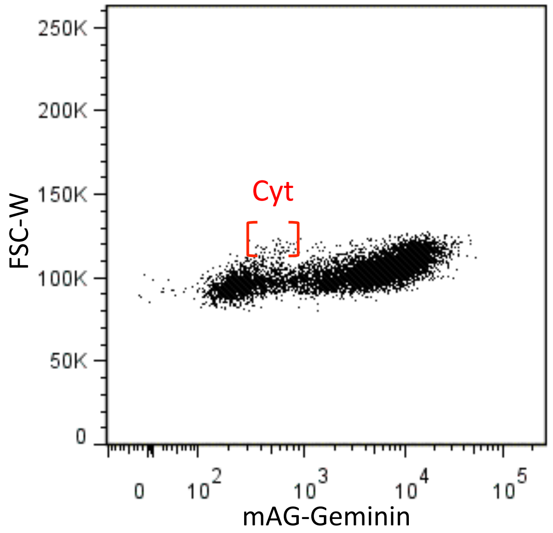

Supplementary Figure 4. ***Gating fixed cytokinetic L1210 cells by FSC-W.*** **(A)** L1210 cells expressing mAG-Geminin were fixed in 4% PFA. A bivariate plot showing mAG-Geminin vs. FSC-W is shown. The Cyt population is indicated in red.

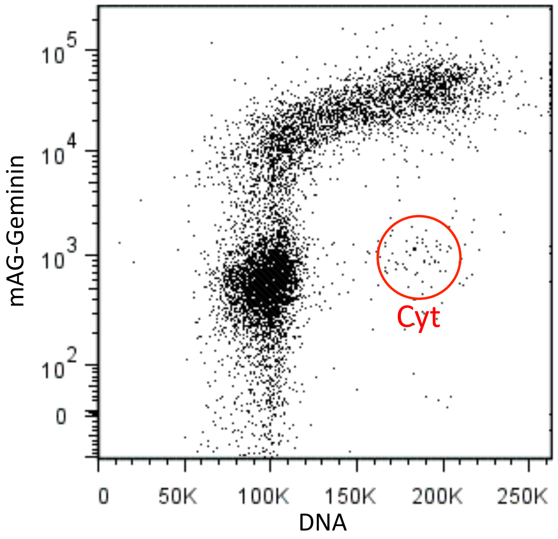

Supplementary Figure 5. ***Gating fixed cytokinetic HeLa cells.*** **(A)** HeLa cells expressing mAG-Geminin were stained with Hoechst 33342 and fixed in 4% PFA. A bivariate plot showing mAG-Geminin vs. DNA is shown. The Cyt population is indicated in red.

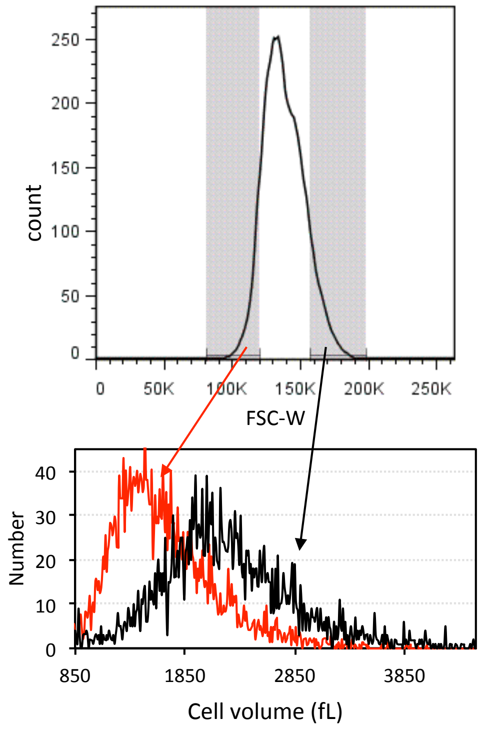

Supplementary Figure 6. ***HeLa cell size approximation by FSC-W.*** HeLa cells showing the lowest (red) or highest (black) 10% FSC-W signals were sorted for direct volume measurements by a Multisizer 4 Coulter Counter®.
